# Genome-wide approach to study gene–nutrient intake interactions in type 2 diabetes mellitus in a large Korean cohort

**DOI:** 10.3389/fnut.2025.1538813

**Published:** 2025-05-07

**Authors:** Jae-Min Park, Ja-Eun Choi, Youhyun Song, Yu-Jin Kwon, Ji-Won Lee, Kyung-Won Hong

**Affiliations:** ^1^Department of Family Medicine, Uijeongbu Eulji Medical Center, Eulji University, Uijeongbu, Republic of Korea; ^2^Department of Medicine, Graduate School of Medicine, Yonsei University, Seoul, Republic of Korea; ^3^Advanced Institute of Technology, Theragen Health Co., Ltd., Seongnam, Republic of Korea; ^4^Department of Family Medicine, Gangnam Severance Hospital, Yonsei University College of Medicine, Seoul, Republic of Korea; ^5^Healthcare Research Team, Health Promotion Center, Gangnam Severance Hospital, Yonsei University College of Medicine, Seoul, Republic of Korea; ^6^Department of Family Medicine, Yongin Severance Hospital, Yonsei University College of Medicine, Yongin, Republic of Korea; ^7^Department of Family Medicine, Severance Hospital, Yonsei University College of Medicine, Seoul, Republic of Korea; ^8^Institute for Innovation in Digital Healthcare, Yonsei University, Seoul, Republic of Korea

**Keywords:** nutrient, single-nucleotide polymorphism, type 2 diabetes mellitus, gene–diet interaction, genome-wide association study

## Abstract

**Introduction:**

A comprehensive understanding of gene–diet interactions is necessary to establish proper dietary guidelines for the prevention and management of type 2 diabetes mellitus (T2DM). We examined the roles of genetic variants and their interactions with T2DM-associated nutrients in a large-scale genome-wide association study of Korean adults.

**Methods:**

A total of 50,808 participants from a Korean genome and epidemiology study were included. Dietary intake was assessed using a food frequency questionnaire. Dietary nutrient intake was classified based on the Korean Dietary Reference Intakes (DRIs). Odds ratio (OR) and 95% confidence interval were calculated after adjusting for age, sex, exercise, smoking, alcohol consumption, total energy consumption, principal component 1, and principal component 2.

**Results:**

Among the individuals consuming vitamin A (retinol equivalent) above the DRI, the carriers of the melanotransferrin (*MELTF*) rs73893755 minor allele (T) had a higher risk of T2DM than the non-carriers (OR = 1.85, *p*-value = 1.66 × 10^−8^; interaction *p*-value = 7.61 × 10^−8^). Similarly, among the individuals consuming cholesterol (mg/day) above the DRI, the carriers of the tripartite motif-containing protein 25 (*TRIM25*) rs139560285 minor allele (A) had a higher risk of T2DM than the non-carriers (OR = 2.02, *p*-value = 4.06 × 10^−8^; interaction *p*-value = 4.28 × 10^−8^).

**Discussion:**

Our results suggest that interactions between specific polymorphisms and certain nutrients may influence T2DM development.

## 1 Introduction

Type 2 diabetes mellitus (T2DM) is a global public health challenge owing to its widespread prevalence and the associated risks of various complications, making it a leading cause of disability and mortality among adults (1, 2). Individuals with T2DM typically incur approximately 2.6 times higher medical costs than those without T2DM (3). The prevalence of T2DM has been on the rise globally, increasing from 4.7% in 1980 to 9.3% in 2019, with projections estimating a further increase to 10.2% (578 million) by 2030 and 10.9% (700 million) by 2045 (4, 5). In South Korea, the prevalence of T2DM has rapidly increased from 8.6% in 2001 to 16.7% in 2020 (6, 7). Currently, T2DM is the sixth leading cause of death in South Korea (8). Thus, identifying high-risk individuals and optimizing interventions are of importance.

Dietary habits, including the excessive intake of certain nutrients, are critical modifiable determinants that can reduce T2DM risk and progression (9). Health authorities have established dietary reference values such as the recommended dietary allowance, adequate intake, tolerable upper intake level, and estimated average requirement to help prevent chronic diseases (10). Previous research has explored the relationship between dietary factors and T2DM incidence by examining diet quality, food groups, specific foods, and various nutrients (11). However, a consensus on the optimal nutritional strategy for patients with T2DM remains to be reached. Therefore, dietary strategies should be personalized according to individual risk factors, eating preferences, and metabolic goals.

Genetic factors substantially influence the susceptibility to T2DM, a polygenic disease with high heritability (12). Genome-wide association studies (GWAS) have identified over 40 loci related to glycemic traits in Korea, including *CDKAL1* (rs7754840), *SLC30A8* (rs13266634), and *TCF7L2* (rs7903146); however, these genetic variations explain less than 10% of T2DM heritability (13). Therefore, the interactions between genetic factors and lifestyle, particularly diet, are subject of intense research on T2DM development (14). Dietary components such as carbohydrates and fiber can influence the effects of specific T2DM-associated genetic variants. For instance, variants such as *ADAMT59* (rs4607103), *CDKN2A/2B* (rs1801282), and *FTO* (rs8050136) in non-Hispanic white people and *ADAMT59* (rs4607103) and *THADA* (rs7578597) in non-Hispanic black people show significant interactions with dietary intake (15). These findings highlight the complex interplay between genetics and diet in T2DM pathogenesis and suggest that personalized dietary recommendations based on genetic profiles could improve T2DM management and prevention. However, most previous studies have focused on a limited number of genetic variants and dietary factors, restricting the comprehensive understanding of these interactions.

Here, we aimed to investigate a broad spectrum of nutrients, including micronutrients, along with gene variability and gene–nutrient interactions in T2DM. Our specific goal was to identify novel genetic variants that influence T2DM through significant gene–nutrient interactions using data from a large-scale GWAS of the Korean population.

## 2 Methods

### 2.1 Study population

**Figure 1:**
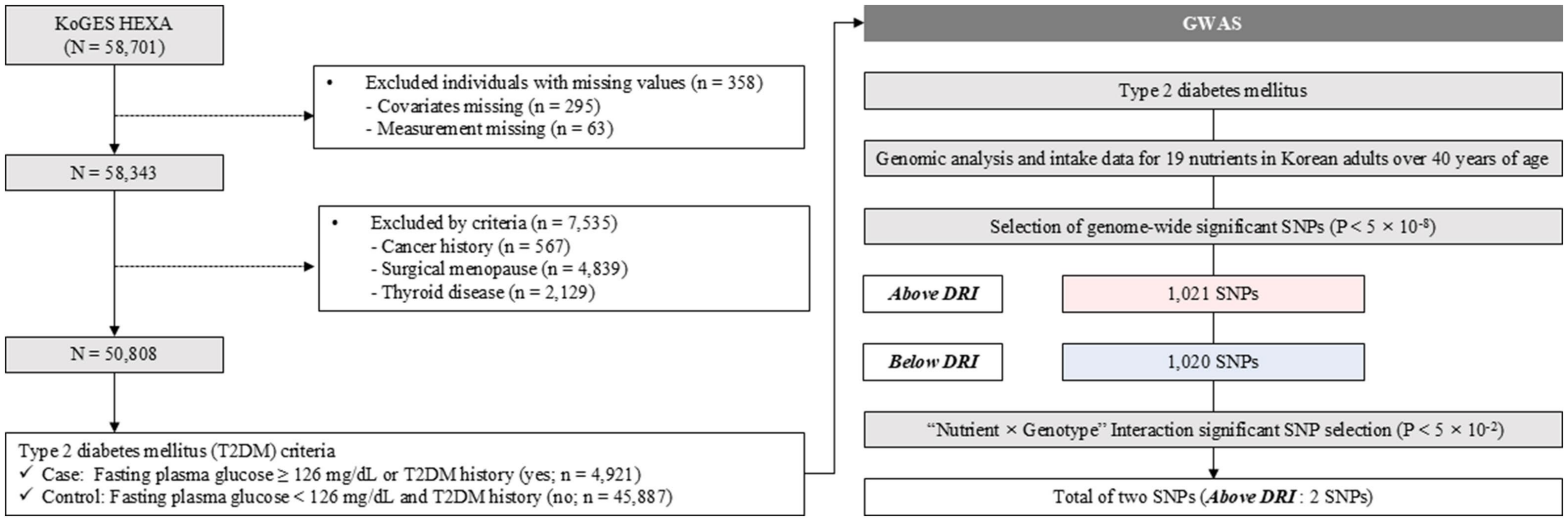
Scheme of the study. GWAS, genome-wide association study; SNP, single-nucleotide polymorphism; DRI, daily reference intake.

The Korean Genome and Epidemiology Study (KoGES) is a government-funded genome epidemiological study platform designed to identify the genetic and environmental etiology of common complex diseases in South Koreans (National Research Institute of Health, Korea Disease Control and Prevention Agency, and the Ministry of Health and Welfare). The KoGES enrolls community dwellers and participants from the national health examinee registry, which includes men and women aged ≥40 years at baseline. Data of 58,701 participants for whom genome-wide single-nucleotide polymorphism (SNP) genotype data were available were included in the KoGES dataset. The GWAS data have been deposited at https://biobank.nih.go.kr/cmm/main/mainPage.do. Researchers can access the GWAS data after obtaining permission from relevant authorities. We excluded 295 samples with missing covariate values, including age, sex, smoking status, drinking status, exercise status, and calories as well as 63 samples with missing values required for T2DM diagnosis, including fasting plasma glucose level and T2DM history. Among them, 7,535 people were excluded from treatment for cancer, thyroid disease, or surgical menopause. Finally, 50,808 participants, 4,921 (9.7%) patients with T2DM and 45,887 (90.3%) controls, were included. A flowchart of the study population is shown in Figure 1. The study protocol adhered to the ethical guidelines of the 1975 Declaration of Helsinki, as reflected in an *a priori* approval by the Institutional Review Board of Theragen Bio (approval number: 700062-20190819-GP-006-02). All participants voluntarily signed an informed consent form before participating in the study.

### 2.2 Genotyping and covariates

The dataset comprises a wide range of phenotypic and environmental measures, biological samples (i.e., serum, plasma, and urine), genome-wide genotype information, and links to health and administrative records. After collecting blood DNA samples according to standard protocols, all samples were transported to the National Biobank of Korea and stored for future research purposes. Genomic DNA was extracted from peripheral blood samples. The extracted genomic DNA was genotyped using Korea Biobank Arrays (KoreanChip). Detailed information regarding KoreanChip has been reported previously (16). We applied the following criteria in the analysis of KoreanChip data to control the quality of genotyping results: call rate > 97%, missing genotype > 0.01, minor allele frequency > 0.01, and Hardy–Weinberg equilibrium (HWE) *p* > 0.000001. HWE was assessed using the standard equation: *p* + *q* = 1 and *p^2^* + *2pq* + *q^2^* = 1, where *p* and *q* represent the frequencies of the dominant and recessive alleles, respectively. Genotype frequencies were calculated as *p^2^* (homozygous dominant), *2pq* (heterozygous), and *q^2^* (homozygous recessive). We applied an HWE filter using the --hwe 0.00001 option in PLINK v1.9 to exclude SNPs that significantly deviated from equilibrium.

Trained technicians performed the anthropometric measurements, with participants wearing light clothes and being barefoot. All measurements were taken to the nearest 0.1 cm in height and 0.1 kg in weight. The body mass index was calculated as weight (kg) divided by height (m) squared. Waist circumference was measured midway between the lowest rib and top of the iliac crest. Samples for the measurement of blood glucose, total cholesterol, triglycerides, and high-density lipoprotein cholesterol levels were obtained after at least 8 h of fasting. Trained interviewers questioned the participants about their lifestyles. Based on smoking status, the participants were categorized into non-smokers, former smokers, and current smokers.

Trained interviewers collected alcohol consumption data using an alcoholic beverage questionnaire. Participants were asked to report their drinking status (current, past, or non-drinkers) and their drinking habits in case of current drinkers, such as the average frequency of drinking during the previous year. To estimate the amount of alcohol consumed by current drinkers, the participants were asked how frequently they consumed each alcoholic beverage and how many drinks they consumed per occasion. Alcohol consumption (g/day) was calculated based on the alcohol content of each alcoholic beverage. According to the questionnaire, regular exercise was defined as routine performance of ≥30-min exercise/day. Systolic blood pressure (SBP) and diastolic blood pressure (DBP) were measured at least twice in the sitting position. Hypertension was defined as SBP ≥ 140 mg/dL or DBP ≥ 90 mg/dL or an intake of any antihypertensive medication. T2DM was defined as fasting serum glucose ≥ 126 mg/dL or a history of the presence of one or more of the following conditions: fasting glucose level ≥ 126 mg/dL, HbA1c ≥ 6.5%, and 2-h plasma glucose level in oral glucose tolerance test ≥ 200 mg/dL, based on the American Diabetes Association, or ongoing treatment with oral antidiabetic medications or insulin therapy.

### 2.3 Determination of nutrition intake reference

To assess the usual dietary intake of the participants, a semi-quantitative food frequency questionnaire (FFQ) including 103 items was developed for the KoGES (17, 18). Participants reported the frequency and amount of food consumed in the past year. Nutritional intake criteria were set based on the 2020 Korean Dietary Reference Intakes (KDRIs; Ministry of Health and Welfare’s Research Project, 2020). The DRI is a general term for a set of reference values used to plan and assess the nutrient intake of healthy people. For macronutrients (carbohydrates, proteins, and fat), an acceptable macronutrient distribution range (AMDR), which is the range of intake for energy sources, was considered. The AMDR for carbohydrates, proteins, and fat was 55–65%, 7–20%, and 15–30%, respectively (19). We determined the upper limit of each macronutrient’s AMDR as the macronutrient intake reference criteria. The DRI for potassium (K), sodium (Na), fiber, and vitamin E was set based on adequate intake, which is the recommended average daily nutrient intake level based on experimentally derived intake levels or approximations of the observed mean nutrient intake considered adequate by a group (or groups) of apparently healthy people (20). The DRI for cholesterol was set based on chronic disease endpoints, which are the reference values for intake that affect chronic disease outcomes (21). The DRI of other nutrients was set based on the recommended nutrient intake, which is the average daily level of nutrient intake sufficient to meet the nutrient requirements of approximately all (97–98%) healthy people. The DRI of nutrients varied according to age and sex. As the KoGES included participants aged 40 years and older, we calculated the mean values of the DRI of the population aged over 40 years according to sex. The specific criteria for DRI are shown in Supplementary Table 1 (22).

### 2.4 Statistical analysis

Data are presented as mean ± standard deviation or number (percentage). To compare differences between the groups based on the presence of DM, we used an independent two-sample *t*-test for continuous variables and a chi-squared test for categorical variables. We performed principal component (PC) analysis to reduce the bias of genomic data according to the region where samples were collected and used PC1 and PC2 as covariates in the statistical analyses. For each nutrient group (above or below the DRI), we conducted a genome-wide scan to identify the specific genetic variants associated with DM. We analyzed the GWAS results for the DM and control groups. All GWAS were conducted using logistic regression analysis after adjusting for age, sex, exercise, smoking, alcohol intake, total energy consumption, PC1, and PC2 using PLINK version 1.9. Significant associations were defined by genome-wide *p* values of <5.00 × 10^−8^. A regional association plot confirmed the surrounding SNPs and nearby genes that were in linkage disequilibrium with the SNPs. For each genome-wide significant region (*p* < 5 × 10^−8^), the lead SNP was defined as the variant with the most significant *p*-value within a ±500 kilobase (kb) window. After selecting the lead SNPs, we also selected unique SNPs that were significant only in the nutrient group, and conducted further interaction analysis using the generalized linear model of R statistics (Version 4.0.3; R Foundation for Statistical Computing, Vienna, Austria). Finally, we selected SNPs with interaction *p*-values < 0.05 as the important SNPs that can significantly interact with nutrients related to disease susceptibility.

To further explore the physiological relevance of the selected SNPs, mean hemoglobin and total cholesterol levels were compared across rs73893755 and rs139560285 genotypes, respectively, within nutrient intake strata (above or below the DRI), using general linear models adjusted for age and sex. In addition, differences in dietary vitamin A and cholesterol intake across genotypes were assessed.

## 3 Results

Table 1 shows the general characteristics of the study population. The mean age ± standard deviation of the total population was 53.6 ± 8.1 years, and 61.4% were women. Of the 50,808 participants, 4,921 (9.7%) had T2DM. The participants with T2DM were older and had a higher body mass index, SBP, DBP, total cholesterol level, and triglyceride level than those without T2DM. The proportions of men and current smokers were greater among participants with T2DM than among those without. The participants with T2DM consumed more alcohol than those without T2DM. Regarding nutritional intake, carbohydrate intake (%) was significantly higher in participants with T2DM. The total calorie intake (kcal/day) and the intake of fat (%), calcium (mg/day), phosphorus (mg/day), iron (mg/day), potassium (mg/day), vitamin B1 (mg/day), vitamin B2 (mg/day), niacin (mg/day), vitamin C (mg/day), zinc (mg/day), vitamin B6 (mg/day), folate (mcg/day), fiber (g/day), vitamin E (mg/day), and cholesterol (mg/day) were significantly lower in participants with T2DM than in those without. The intake of protein (%), vitamin A (retinol equivalent; R.E.), and sodium (mg/day) was not significantly different between the groups.

**Table 1:** Characteristics of the study population.

Variables	Total	With diabetes	Without diabetes	*P*-value
*N*	50,808	4,921	45,887	
Age, years	53.6 ± 8.1	57.8 ± 7.5	53.1 ± 8.1	<0.001
Sex, *n* (%)				<0.001
Male	19,595 (38.6)	2,661 (54.1)	16,934 (36.9)	
Female	31,213 (61.4)	2,260 (45.9)	28,953 (63.10)	
Smoking status, *n* (%)				<0.001
None	35,796 (70.5)	2,811 (57.2)	32,985 (71.9)	
Former smoker	8,806 (17.3)	1,279 (26.0)	7,527 (16.4)	
Current	6,170 (12.2)	825 (16.8)	5,345 (11.7)	
Alcohol intake, g/day	57.2 ± 176.4	77.7 ± 188.1	55.01 ± 175.0	<0.001
Regular exerciser, *n* (%)	27,672 (54.4)	2,852 (58.0)	24,820 (54.1)	<0.001
Body mass index, kg/m^2^	23.9 ± 2.9	25.1 ± 3.1	23.77 ± 2.8	<0.001
Waist circumference, cm	80.9 ± 9.0	85.6 ± 9.0	80.4 ± 8.9	<0.001
Systolic blood pressure, mmHg	122.5 ± 15.0	127.4 ± 15.2	121.9 ± 14.9	<0.001
Diastolic blood pressure, mmHg	75.8 ± 9.9	77.4 ± 9.7	75.6 ± 9.9	<0.001
Glucose, mg/dL	95.3 ± 19.9	133.4 ± 39.2	91.1 ± 9.8	<0.001
Total cholesterol, mg/dL	197.0 ± 35.6	188.21 ± 40.5	197.9 ± 34.9	<0.001
Triglycerides, mg/dL	126.0 ± 86.7	160.5 ± 121.8	122.3 ± 81.1	<0.001
High-density lipoprotein cholesterol, mg/dL	53.5 ± 13.1	48.8 ± 11.9	54.0 ± 13.2	<0.001
Total energy, kcal/day	1,738.2 ± 570.4	1,699.9 ± 558.8	1,742.3 ± 571.5	<0.001
Carbohydrate (%)	71.0 ± 10.0	71.3 ± 10.6	70.9 ± 10.0	0.006
Protein (%)	13.2 ± 3.2	13.2 ± 3.4	13.2 ± 3.2	ns
Fat (%)	13.8 ± 5.8	13.1 ± 5.9	13.9 ± 5.8	<0.001
Calcium, mg/day	438.7 ± 256.2	415.6 ± 246.0	441.2 ± 257.1	<0.001
Phosphorus, mg/day	883.9 ± 359.3	860.4 ± 351.4	886.4 ± 360.0	<0.001
Iron, mg/day	9.83 ± 5.12	9.59 ± 5.11	9.86 ± 5.12	<0.001
Potassium, mg/day	2,210.9 ± 1,045.1	2,118.1 ± 1012.3	2,220.9 ± 1048.1	<0.001
Vitamin A (R.E.)	473.9 ± 340.0	465.1 ± 341.1	474.8 ± 339.9	ns
Na, mg/day	2,431.2 ± 1395.9	2,416.9 ± 1432.4	2,432.7 ± 1391.9	ns
Vitamin B1, mg/day	0.92 ± 1.00	0.88 ± 1.06	0.93 ± 0.99	0.001
Vitamin B2, mg/day	0.82 ± 0.99	0.76 ± 1.01	0.83 ± 0.98	<0.001
Niacin, mg/day	14.36 ± 6.47	13.96 ± 6.30	14.41 ± 6.49	<0.001
Vitamin C, mg/day	104.1 ± 67.0	96.7 ± 61.4	104.9 ± 67.5	<0.001
Zinc, mg/day	7.84 ± 3.90	7.68 ± 3.64	7.86 ± 3.92	0.001
Vitamin B6, mg/day	1.50 ± 1.17	1.44 ± 1.22	1.50 ± 1.16	<0.001
Folate, mcg/day	214.2 ± 120.1	206.1 ± 117.3	215.0 ± 120.3	<0.001
Fiber, g/day	5.58 ± 3.19	5.41 ± 3.06	5.60 ± 3.10	<0.001
Vitamin E, mg/day	7.99 ± 4.62	7.59 ± 4.65	8.03 ± 4.62	<0.001
Cholesterol, mg/day	168.6 ± 124.5	154.6 ± 119.9	170.1 ± 124.9	<0.001

R.E., retinol equivalent.

Data are presented as mean ± standard deviation for continuous variables or number (%) for categorical variables.

*p-values were obtained via independent two-sample t-test for continuous variables and chi-squared test for categorical variables.*

Figure 1 illustrates the overall study scheme. Nineteen target nutrients were investigated during the KoGES. A total of 50,808 participants were included in the GWAS and subsequent analyses, categorized into T2DM cases (*n* = 4,921) and controls (*n* = 45,887). After the initial GWAS, 2,041 SNPs (1,021 SNPs above and 1,020 SNPs below the DRI) showed genome-wide significance. Among these significant SNPs, we identified two SNPs related to T2DM in the DRI groups, with significant nutrition-by-gene interactions.

**Figure 2:**
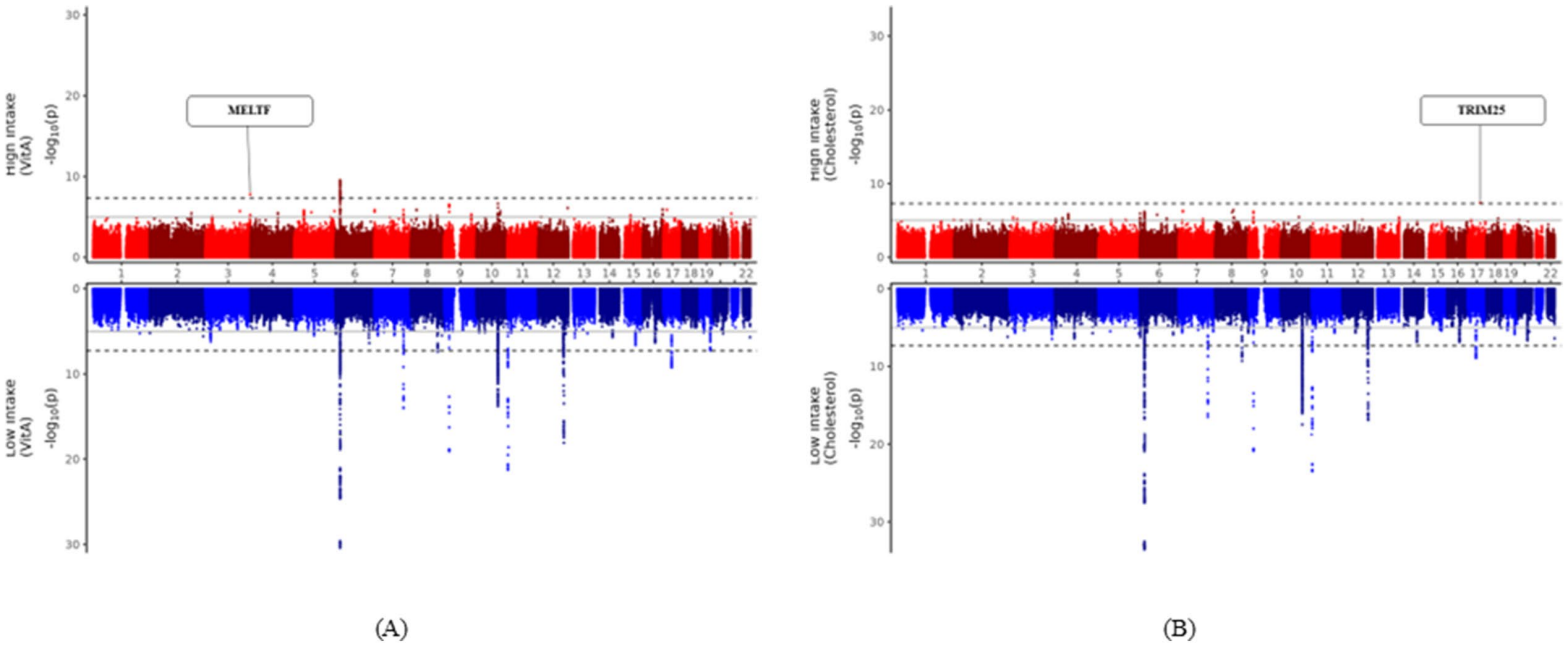
Miami plots presenting the results of the GWAS. Miami plots presenting the results of the GWAS on the risk of T2DM prevalence in the high nutrient-intake group (red graph) and low nutrient-intake group (blue graph). The horizontal axis of each graph is the SNP position from chromosomes 1 to 22 and the vertical axis is the −log_10_ transformation of the statistical significance (*p*-value) for T2DM. The solid line in the graph is the genome-wide suggestive level (*p*-value < 1 × 10^−5^) and the dotted line is the genome-wide significance level (*p*-value < 5 × 10^−8^). **(A)** Miami plot highlighting the *MELTF* region that is specifically significant in the vitamin A high-intake group. **(B)** Miami plot highlighting the *TRIM25* region that is specifically significant in the cholesterol high-intake group. GWAS, genome-wide association study; SNP, single-nucleotide polymorphism.

**Figure 3:**
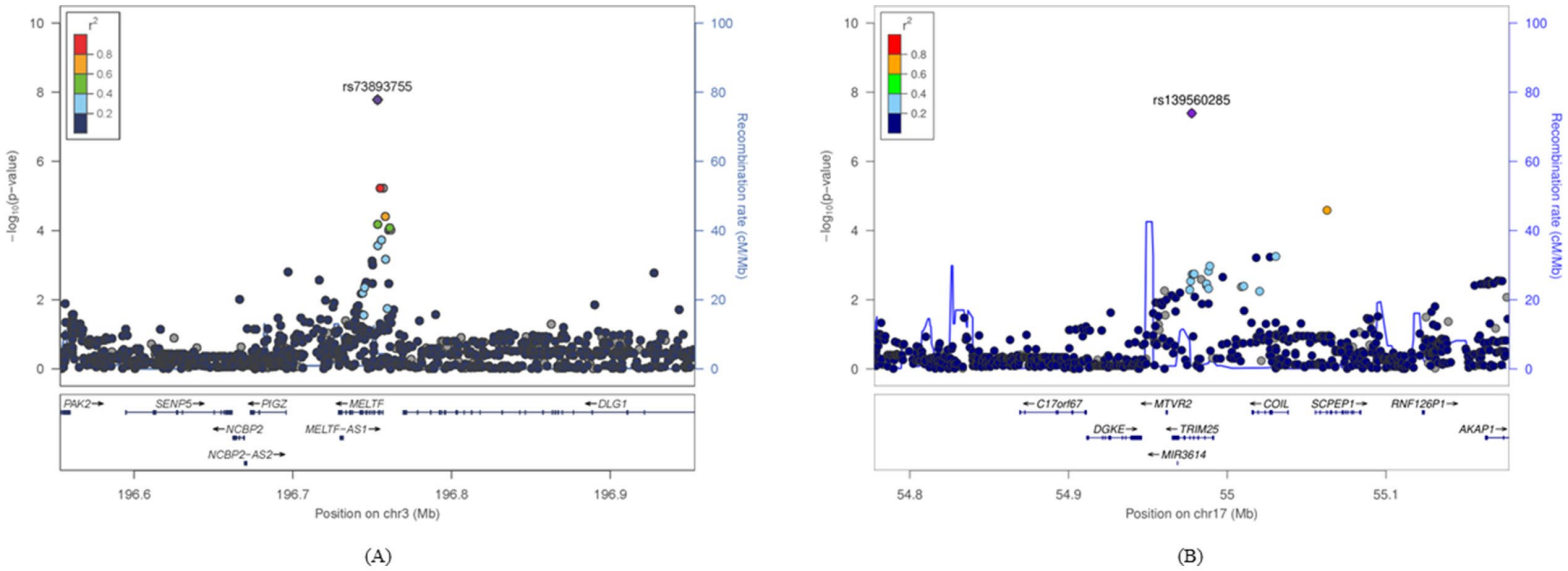
Signal plots for the statistically significant SNP regions confirmed in a specific diet group. The lead SNPs identified in the GWAS and the ±400-kbp region were plotted. The horizontal axis represents the base pair position on the chromosome where the SNPs are located and the left vertical axis represents the *p*-value transformed into −log10. The vertical axis on the right represents the region where recombination can occur during cell division. **(A)** The genomic region around rs3893755 in the group with high vitamin A intake and **(B)** the genomic region around rs139560285 in the group with high cholesterol intake. GWAS, genome-wide association study; SNP, single-nucleotide polymorphism.

Two SNPs were identified as uniquely significant (i.e., not showing widespread significance over nutrients and showing unilateral significance either above or below the DRI) in each of the two nutrient groups (cholesterol and vitamin A). Results of the two significant SNPs are depicted in Figure 2 as Miami plots using log_10_ transformed *p*-values. Figure 3 shows the surrounding association signals and genes located in the lead SNP region at the positions of the two SNPs identified. A few SNPs showed a significant association with T2DM, located around the SNP rs73893755, with the functional gene at that location of melanotransferrin (*MELTF*; Figure 3A). There were relatively few SNPs with a significant association with T2DM around rs139560285; however, tripartite motif-containing protein 25 (*TRIM25*) was at that location (Figure 3B). GWAS and gene–nutrient interaction analysis results are shown in Supplementary materials 1, 2.

Table 2 presents SNPs with significant nutrient-by-gene interactions associated with T2DM. Two gene-by-nutrient interactions were associated with T2DM prevalence. Among the individuals consuming vitamin A (R.E.) above the DRI, carriers of the *MELTF* rs73893755 minor allele (T) had a higher risk of T2DM than the non-carriers (odds ratio [OR] = 1.85, *p*-value = 1.66 × 10^−8^; interaction *p*-value = 7.61 × 10^−8^). Similarly, among the individuals consuming cholesterol (mg/day) above the DRI, carriers of the *TRIM25* rs139560285 minor allele (A) had a higher risk of T2DM than the non-carriers (OR = 2.02, *p*-value = 4.06 × 10^−8^; interaction *p*-value = 4.28 × 10^−8^).

**Table 2:** Single-nucleotide polymorphisms (SNPs) showing significant interactions with nutrients associated with diabetes.

Chr	SNP	BP	Positional candidate gene	Alleles		Alternative allele frequency	Other Ethnicities			Coding allele	Coding allele frequency	Nutrient Intake	Gene association result		Gene and nutrient association results		
				Reference	Alternative		EAS	EUR	AMR				OR (95% CI)	*P*-value	OR (95% CI)	Interaction *P* value	Top *P* value
3	rs73893755	196753267	*MELTF*	C	T	0.043	0.038	0	0.033	T	0.043	Above Vitamin A	1.08 (0.97–1.19)	0.168	1.85 (1.61–2.77)	7.61 × 10^−8^	1.66 × 10^−8^
17	rs139560285	54977570	*TRIM25*	G	A	0.059	0.047	0	0.003	A	0.051	Above Cholesterol	1.02 (0.93–1.13)	0.628	2.02 (1.73–3.17)	4.28 × 10^−8^	4.06 × 10^−8^

AMR, American; BP, base pair; Chr, chromosome; EAS, East Asian; EUR, European; OR, odds ratio; CI, confidence interval.

*p*-value was calculated using logistic regression analysis adjusted for age, sex, exercise, smoking, alcohol intake (g/day), total calorie consumption, PC1, and PC2.

As an indirect indicator of iron status, we further examined hemoglobin levels according to rs73893755 genotypes stratified by vitamin A intake level (Supplementary Table 2). Hemoglobin levels decreased with the T allele among individuals consuming vitamin A above the DRI, while the opposite trend was observed in those below the DRI, suggesting a potential gene–nutrient interaction. Similarly, to explore the physiological relevance of the TRIM25 rs139560285 variant, we assessed total cholesterol levels across genotypes stratified by dietary cholesterol intake (Supplementary Table 3). No statistically significant differences were observed. These results suggest that the observed gene–diet interaction associated with T2DM risk may not be directly explained by circulating cholesterol levels.

## 4 Discussion

Nutrition can influence T2DM development by directly altering the expression of genes involved in critical metabolic pathways or indirectly affecting the incidence of genetic mutations at the base sequence or chromosomal level (23). The present study showed that certain dietary factors interact with genetic variants in T2DM. We identified significant interactions between vitamin A and the rs73893755 variant near the *MELTF* region in those who consumed vitamin A above the DRI. A similar significant interaction was noted for the genetic variant rs139560285 near the *TRIM25* region in those who consumed cholesterol above the DRI. Interestingly, there was no significant difference in the T2DM risk attributed to these genetic variants when nutrition was not considered.

Several studies have examined gene–diet interactions in T2DM using the genetic risk score (GRS). The Airwave Health Monitoring study of 3,733 white British participants reported a significant interaction between the GRS and whole grain intake (*P* for interaction = 0.04). The effect of gene–diet (whole-grain) interactions on HbA1c level was greater in high-genetic-risk individuals (24). In a study using data from the UK Biobank, there was a negative interaction of blood HbA1c with the GRS and diet quality score. A higher diet quality score is associated with a greater reduction in T2DM risk among individuals with a higher genetic risk (25). Fruit intake interacts with the T2DM-GRS (*P* for interaction = 0.04) and related glucose metabolic traits (*P* for interaction ≤ 0.03). The association between fruit intake and a lower T2DM risk is more prominent in populations with a stronger genetic predisposition to T2DM (25). Here, we utilized nutritional reference values based on DRI to plan and evaluate nutrient intake in healthy individuals, considering potential deficiencies, inadequacies, and toxicities. We assessed a wide range of nutrient and gene variabilities using a comprehensive approach. Consequently, we identified novel SNPs that significantly interacted with specific nutrients related to T2DM.

Individuals with the *MELTF* variant rs73893755 who consumed vitamin A above the DRI level were found to have a higher risk of T2DM. The exact mechanisms underlying this association remain unclear. Nevertheless, it should be noted that high iron levels are a well-known risk factor for T2DM (26), and iron overload related to vitamin A consumption may play a critical pathophysiological role in this context. Vitamin A influences iron metabolism, including iron absorption and erythropoiesis (27, 28). *MELTF* encodes a protein similar to transferrin, which is involved in iron transport and metabolism (29, 30). Therefore, excessive vitamin A intake can lead to increased iron absorption, potentially overwhelming the regulatory capacity of *MELTF* and elevating T2DM risk due to iron overload.

The second main finding of our study is the interaction between cholesterol intake and an SNP in *TRIM25* (rs139560285) contributing to T2DM risk. Individuals carrying rs139560285 who consumed cholesterol above the DRI had a higher risk of T2DM, suggesting that such individuals could be more vulnerable to T2DM when consuming more dietary cholesterol. Dietary cholesterol is abundant in various foods such as egg yolk, red meat, butter, and animal viscera. Although the relationship between cholesterol consumption and T2DM is still debated, studies have suggested a positive dose–response relationship (31, 32). One potential mechanism linking cholesterol intake to T2DM is chronic low-grade inflammation, which plays a role in disease pathogenesis (33). Dietary cholesterol exacerbates obesity-induced macrophage accumulation in adipose tissue, contributing to systemic inflammation (34). It also increases the levels of serum amyloid A, an inflammatory marker, activating pro-inflammatory signaling cascades upon consumption (35, 36). These findings suggest that excessive cholesterol intake significantly affects inflammation-induced diabetes. TRIM25 is a crucial regulator of lipid metabolism by suppressing adipocyte differentiation and a regulator of glucose metabolism by decreasing the expression of tricarboxylic acid cycle enzymes (37, 38). TRIM25 also plays a role in modulating inflammatory responses. TRIM25 has been reported to enhance NF-κB activation; moreover, it can directly regulate TNF-*α*-induced NF-κB signaling, thereby stimulating the production of pro-inflammatory cytokines such as IL-1β and TNF-α (39–41). This finding suggests that the interaction between cholesterol intake and TRIM25 variants may affect T2DM development through chronic low-grade inflammation.

Higher cholesterol intake is generally associated with greater consumption of animal-derived foods, including red meat, organ meats, and egg yolks (42). Likewise, higher vitamin A intake may reflect frequent consumption of liver, eggs, and full-fat dairy products, which are rich sources of preformed vitamin A (43). These patterns suggest that controlling specific food groups may benefit T2DM prevention efforts, especially in individuals with susceptible genotypes.

Although direct iron biomarkers were not available, hemoglobin concentration was analyzed as a proxy for iron status. Hemoglobin levels showed opposite trends across rs73893755 genotypes depending on vitamin A intake level: they decreased with T allele dosage in the high-intake group but increased slightly in the low-intake group. This pattern supports the possibility of a gene–nutrient interaction between *MELTF* and vitamin A influencing iron metabolism and T2DM risk. Furthermore, while vitamin A intake did not differ significantly across genotypes in the high-intake group, a modest increase was observed among T allele carriers in the low-intake group, providing partial biological plausibility for the observed interaction. In contrast, total cholesterol levels did not differ significantly across rs139560285 genotypes, regardless of dietary cholesterol intake. Given that the primary aim of this study was to examine interactions between dietary cholesterol and genetic variation, the absence of significant differences in circulating cholesterol levels suggests that other biological mechanisms may underlie the observed association with T2DM. Genetic influences on T2DM risk may not necessarily be mediated through serum cholesterol levels, particularly within the context of complex metabolic regulation.

These findings have potential implications for personalized nutrition in the Korean population. As interactions between specific genetic variants and dietary components may influence T2DM risk, incorporating genetic screening alongside dietary assessment could enhance the effectiveness of prevention and management strategies. For example, individuals with risk alleles that exhibit adverse responses to high cholesterol or vitamin A intake may benefit from more targeted nutritional guidance. In the context of Korea’s rapidly aging population and rising diabetes prevalence, such gene-informed approaches may support more precise public health interventions. Our results are consistent with the emerging field of nutrigenetics and precision nutrition, which seek to integrate genomic information into individualized dietary recommendations for chronic disease prevention (44, 45). Given the population-specific nature of dietary patterns and genetic profiles, gene–diet interaction studies in Koreans are particularly valuable for developing culturally appropriate personalized interventions for T2DM.

There were some limitations to consider when interpreting our findings. First, our study was conducted exclusively in Korean adults aged 40–69 years, necessitating further research in other ethnic groups or countries for broader applicability. Second, most variables were self-reported, which may have affected accuracy due to recall bias. Although the FFQ method used in the KoGES is reliable and validated, it is prone to inaccuracies in absolute nutrient values and lacks specificity for food details (46). Additionally, T2DM results from complex interactions between genetic susceptibility and various environmental factors (47). Therefore, gene–nutrient interactions alone cannot fully explain T2DM development, necessitating comprehensive approaches to understand the multifactorial nature of the disease.

Nevertheless unlike most previous studies that focused on the relationship between specific genetic variants and a limited number of dietary patterns, our study comprehensively evaluated a wide range of nutrients, including micronutrients and other minor dietary factors, in addition to genetic variability. Such an approach has not been previously employed. Additionally, this study provides novel evidence of gene–nutrient interactions in T2DM based on the comprehensive GWAS of a large, representative real-world database of the Korean population.

Understanding gene–nutrient interactions based on genotype allows for tailored dietary advice to optimize individual nutrient intake. Although dietary reference values are designed for the general population, the biological “dose” may vary among individuals due to genetic variability affecting the absorption, distribution, elimination, biotransformation, and metabolism of nutrients (23). These genetic differences can significantly influence nutrient processing in the body. Therefore, personalized dietary recommendations can be implemented, potentially improving health outcomes and preventing T2DM more effectively.

Our GWAS provides novel insights into gene–nutrient interactions in T2DM. We found that dietary vitamin A and cholesterol intake above the DRI significantly interacted with T2DM with genome-wide significance. These results suggest that the accumulation of specific polymorphisms at specific loci may influence T2DM development in carriers exposed to certain types and amounts of nutrients.

## Data Availability

Publicly available datasets were analyzed in this study. This data can be found: https://www.kdca.go.kr/contents.es?mid=a40504010000.
