# Supplementary material for: Genome-wide approach to study gene–nutrient intake interactions in type 2 diabetes mellitus in a large Korean cohort
Source: Front Nutr. 2025 May 7;12:1538813. doi: 10.3389/fnut.2025.1538813 (PMC12092224; doi:10.3389/fnut.2025.1538813)
Supplement: Supplementary file 1 [file Table_1.docx]

Supplementary Material

# Supplementary Table

**Supplementary Table 1.** Reference nutrient criteria and distribution in the analyzed Korean adult population

| Nutrient | Men | | |  | Women | | |
| --- | --- | --- | --- | --- | --- | --- | --- |
|  | Reference | Above Group, n (%) | Below Group n (%) |  | Reference | Above Group, n (%) | Below Group n (%) |
| CHO, % | 65 | 16,241 (83.5) | 3,210 (16.5) |  | 65 | 26,304 (85.0) | 4,642 (15.0) |
| Protein, % | 20 | 376 (1.9) | 19,075 (98.1) |  | 20 | 614 (2.0) | 30,332 (98.0) |
| Fat, % | 30 | 202 (1.0) | 19,249 (99.0) |  | 30 | 278 (0.9) | 30,668 (99.1) |
| Ca, mg/day | 737.5 | 1,664 (8.6) | 17,787 (91.4) |  | 775 | 2,756 (8.9) | 28,190 (91.9) |
| P, mg/day | 700 | 14,341 (73.7) | 5,110 (26.3) |  | 700 | 20,992 (67.8) | 9,954 (32.2) |
| Iron, mg/day | 9.5 | 9,175 (47.2) | 10,276 (52.8) |  | 9.25 | 14,484 (46.8) | 16,462 (53.2) |
| K, mg/day | 3500 | 1772 (9.1) | 17,679 (90.9) |  | 3500 | 2,882 (9.3) | 28,064 (90.7) |
| Vitamin A, R.E. | 737.5 | 2,884 (14.8) | 16,567 (85.2) |  | 612.5 | 6,674 (21.6) | 24,272 (78.4) |
| Na, mg/day | 1350 | 16,112 (82.8) | 3,339 (17.2) |  | 1,350 | 24,104 (77.9) | 6,842 (22.1) |
| Vitamin B1, mg/day | 1.15 | 6,416 (33.0) | 13,035 (67.0) |  | 1 | 11,624 (37.6) | 19,322 (62.4) |
| Vitamin B2, mg/day | 1.425 | 1,971 (10.1) | 17,480 (89.9) |  | 1.125 | 6,784 (21.9) | 24,162 (78.1) |
| Niacin, mg/day | 14.75 | 8,890 (45.7) | 10,561 (54.3) |  | 13.25 | 14,840 (48.0) | 16,106 (52.0) |
| Vitamin C, mg/day | 100 | 7,601 (39.1) | 11,850 (60.9) |  | 100 | 14,327 (46.3) | 16,619 (53.7) |
| Zinc, mg/day | 9.5 | 5,199 (26.7) | 14,252 (73.3) |  | 7.5 | 13,528 (43.7) | 17,418 (56.3) |
| Vitamin B6, mg/day | 1.5 | 9,716 (50.0) | 9735 (50.0) |  | 1.4 | 16,318 (52.7) | 14,628 (47.3) |
| Folate, mcg/day | 400 | 1,188 (6.1) | 18,263 (93.9) |  | 400 | 2,056 (6.6) | 28,890 (93.4) |
| Fiber, g/day | 27.5 | 8 (0.04) | 19,443 (99.96) |  | 20 | 88 (0.3) | 30,858 (99.7) |
| Vitamin E, mg/day | 12 | 2,700 (13.9) | 16,751 (86.1) |  | 12 | 4,076 (13.2) | 26,870 (86.8) |
| Cholesterol, mg/day | 300 | 2,361 (12.1) | 17,090 (87.9) |  | 300 | 3,639 (11.8) | 27,307 (88.2) |
| CHO, carbohydrate; K, potassium; P, phosphorus; R.E, retinol equivalents; Na, sodium.  Reference values of nutrients were based on the following criteria by sex.  Acceptable macronutrient distribution range; carbohydrate (%), protein (%), and fat (%).  Recommended nutrient intake; Ca, P, Iron, K, Vitamin A, B1, B2, C, B6, niacin, folate, and zinc.  Adequate intake; K, Na, fiber, and vitamin E.  Cholesterol intake was set based on chronic disease endpoints. | | | | | | | |

**Supplementary Table 2.** Hemoglobin levels across rs73893755 genotypes, stratified by vitamin A intake level

|  | Vitamin A intake | | *P*^1^ | Individuals consuming vitamin A above the DRI | | | *P*^2^ | Individuals consuming vitamin A below the DRI | | | *P*^3^ |
| --- | --- | --- | --- | --- | --- | --- | --- | --- | --- | --- | --- |
|  |  |  |  | rs73893755 | | |  | rs73893755 | | |  |
|  | Above the DRI | Below the DRI |  | CC | CT | TT |  | CC | CT | TT |  |
| N | 9558 | 40839 |  | 8800 | 740 | 18 |  | 37383 | 3397 | 59 |  |
| Age, years | 53.0 ± 8.0 | 53.7 ± 8.1 | <0.001 | 53.1 ± 8.0 | 52.7 ± 7.7 | 53.5 ± 8.7 | 0.535 | 53.7 ± 8.1 | 53.7 ± 8.1 | 54.1 ± 6.9 | 0.883 |
| Sex, n (%) |  |  | <0.001 |  |  |  | 0.267 |  |  |  | 0.232 |
| Male | 2884 (30.2) | 16567 (40.6) |  | 2675 (30.4) | 204 (27.6) | 5 (27.8) |  | 15125 (40.5) | 1414 (41.6) | 28 (47.5) |  |
| Female | 6674 (69.8) | 24272 (59.4) |  | 6125 (69.6) | 536 (72.4) | 13 (72.2) |  | 22258 (59.5) | 1983 (58.4) | 31 (52.5) |  |
| Vitamin A intake (R.E.) | 996.0 ± 426.5 | 356.6 ± 146.1 | <0.001 | 996.6 ± 429.3 | 990.4 ± 396.0 | 923.7 ± 279.4 | 0.854 | 356.5 ± 146.2 | 356.7 ± 144.8 | 407.6 ± 145.9 | 0.042 |
| Hemoglobin levels (g/dL) | 13.78 ± 1.47 | 13.98 ± 1.50 | <0.001 | 13.79 ± 1.46 | 13.77 ± 1.56 | 13.66 ± 1.30 | <0.001 | 13.98 ± 1.50 | 14.00 ± 1.52 | 14.09 ± 1.56 | <0.001 |
| R.E., retinol equivalent.  Data are presented as mean ± standard deviation for continuous variables or number (%) for categorical variables.  *P* values were obtained via independent two-sample *t*-test or one-way or one-way analysis of variance for continuous variables and chi-squared test for categorical variables.  All p-values for the comparison of hemoglobin levels were calculated by adjusting for age and gender.  *P values represent comparisons between high and low vitamin A intake groups (P****¹****), rs73893755 genotypes within the high intake group (P****²****), and rs73893755 genotypes within the low intake group (P****³****).* | | | | | | | | | | | |

**Supplementary Table 3.** Total cholesterol levels across rs139560285 genotypes, stratified by cholesterol intake level

|  | Cholesterol intake | | *P*^1^ | Individuals consuming cholesterol above the DRI | | | *P*^2^ | Individuals consuming cholesterol below the DRI | | | *P*^3^ |
| --- | --- | --- | --- | --- | --- | --- | --- | --- | --- | --- | --- |
|  |  |  |  | rs139560285 | | |  | rs139560285 | | |  |
|  | Above the DRI | Below the DRI |  | GG | GA | AA |  | GG | GA | AA |  |
| N | 6000 | 44397 |  | 5424 | 560 | 16 |  | 40000 | 4289 | 108 |  |
| Age, years | 52.1 ± 8.2 | 53.3 ± 8.1 | <0.001 | 52.4 ± 8.1 | 52.3 ± 8.3 | 53.8 ± 9.3 | 0.264 | 53.8 ± 8.1 | 53.7 ± 8.0 | 53.1 ± 8.4 | 0.622 |
| Sex, n (%) |  |  | 0.206 |  |  |  | 0.347 |  |  |  | 0.228 |
| Male | 2361 (39.3) | 17090 (38.5) |  | 2120 (39.1) | 233 (41.6) | 8 (50.0) |  | 15125 (40.5) | 1414 (41.6) | 28 (47.5) |  |
| Female | 3639 (60.7) | 27307 (61.5) |  | 3304 (60.9) | 327 (58.4) | 8 (50.0) |  | 22258 (59.5) | 1983 (58.4) | 31 (52.5) |  |
| Cholesterol intake, mg/dL | 415.6 ± 156.8 | 136.9 ± 69.9 | <0.001 | 415.9 ± 158.7 | 413.6 ± 137.7 | 405.5 ± 132.3 | 0.928 | 136.7 ± 69.9 | 138.9 ± 69.6 | 134.6 ± 72.3 | 0.156 |
| Total cholesterol level, mg/dL | 198.81 ± 35.55 | 196.76 ± 35.61 | <0.001 | 198.82 ± 35.63 | 198.52 ± 34.25 | 205.06 ± 53.45 | 0.831 | 196.75 ± 35.62 | 196.92 ± 35.50 | 196.81 ± 37.93 | 0.881 |
| Data are presented as mean ± standard deviation for continuous variables or number (%) for categorical variables.  *P* values were obtained via independent two-sample *t*-test or one-way or one-way analysis of variance for continuous variables and chi-squared test for categorical variables.  All p-values for the comparison of hemoglobin levels were calculated by adjusting for age and gender.  *P values represent comparisons between high and low vitamin A intake groups (P****¹****), rs73893755 genotypes within the high intake group (P****²****), and rs73893755 genotypes within the low intake group (P****³****).* | | | | | | | | | | | |
